# Effect of Bronchoscopy on Gas Exchange and Respiratory Mechanics in Critically Ill Patients With Atelectasis: An Observational Cohort Study

**DOI:** 10.3389/fmed.2018.00301

**Published:** 2018-11-13

**Authors:** Kim M. G. Smeijsters, Ronald M. Bijkerk, Johannes M. A. Daniels, Peter M. van de Ven, Armand R. J. Girbes, Leo M. A. Heunks, Jan Jaap Spijkstra, Pieter R. Tuinman

**Affiliations:** ^1^Department of Intensive Care, Amsterdam University Medical Centers, Vrije Universiteit Amsterdam, Amsterdam, Netherlands; ^2^Department of Anesthesiology, Amsterdam University Medical Centers, Vrije Universiteit Amsterdam, Amsterdam, Netherlands; ^3^Department of Anesthesiology, Noordwest Ziekenhuisgroep, Alkmaar, Netherlands; ^4^Department of Pulmonary Diseases, Amsterdam University Medical Centers, Vrije Universiteit Amsterdam, Amsterdam, Netherlands; ^5^Department of Epidemiology and Biostatistics, Amsterdam University Medical Centers, Vrije Universiteit Amsterdam, Amsterdam, Netherlands; ^6^Research VUmc Intensive Care (REVIVE), Amsterdam University Medical Centers, Vrije Universiteit, Amsterdam, Netherlands

**Keywords:** bronchoscopy, atelectasis, intensive care, critical care, gas exchange, respiratory mechanics

## Abstract

**Background:** Atelectasis frequently develops in critically ill patients and may result in impaired gas exchange among other complications. The long-term effects of bronchoscopy on gas exchange and the effects on respiratory mechanics are largely unknown.

**Objective:** To evaluate the effect of bronchoscopy on gas exchange and respiratory mechanics in intensive care unit (ICU) patients with atelectasis.

**Methods:** A retrospective, single-center cohort study of patients with clinical indication for bronchoscopy because of atelectasis diagnosed on chest X-ray (CXR).

**Results:** In total, 101 bronchoscopies were performed in 88 ICU patients. Bronchoscopy improved oxygenation (defined as an increase of PaO_2_/FiO_2_ ratio > 20 mmHg) and ventilation (defined as a decrease of > 2 mmHg in partial pressure of CO_2_ in arterial blood) in 76 and 59% of procedures, respectively, for at least 24 h. Patients with a low baseline value of PaO_2_/FiO_2_ ratio and a high baseline value of PaCO_2_ were most likely to benefit from bronchoscopy. In addition, in intubated and pressure control ventilated patients, respiratory mechanics improved after bronchoscopy for up to 24 h. Mild complications, and in particular desaturation between 80 and 90%, were reported in 13% of the patients.

**Conclusions:** In selected critically ill patients with atelectasis, bronchoscopy improves oxygenation, ventilation, and respiratory mechanics for at least 24 h.

## Background

In critically ill patients, mechanical ventilation might cause ventilator-induced lung injury and hospital-acquired pneumonia, both conditions promote atelectasis and stagnant secretions that may worsen oxygenation and delay weaning from ventilator (1, 2).

Atelectasis may result from numerous causes, for example, from congestion of mucus in the central airways, from increased sputum production, from decreased mucociliary clearance, from decreased cough effectiveness, from increased sputum viscosity, or by a combination of these factors. Treatment of atelectasis in intensive care unit (ICU) patients has been focused on blind airway suctioning, bronchoscopy with or without adjuncts such as nebulization of N-acetylcysteine, and chest physiotherapy. Bronchoscopy is regarded as an attractive method for endobronchial mucus clearing, which possibly results in a more effective airway clearance as it is performed under direct visualization of the airways.

However, the current literature on the effectiveness of bronchoscopy in the treatment of atelectasis is limited. A systematic review concluded that bronchoscopy could be effective in the treatment of atelectasis. The success rates (defined as radiographic improvement on chest X-ray [CXR] or an improved PaO_2_/PAO_2_ ratio) in the ICU patient population had, however, a remarkably wide range of 19–89% (3). Moreover, the effect of bronchoscopy on lung mechanics is largely unknown, which may be of importance in reducing the work of breathing. Furthermore, the superiority of bronchoscopy over blind airway suctioning on clinical relevant endpoints, such as gas exchange, has also not been established (4).

The lack of consistency in the study design in previous case studies (3–11), such as small groups, large variation in the study population, wide range of success, and studies not looking specifically at atelectasis, represents important limitations in the current scientific evidence about bronchoscopy in ICU patients. As such, additional studies are needed to explore further the role of bronchoscopy in atelectasis in this group of patients.

We have hypothesized that bronchoscopy for atelectasis in the ICU improves gas exchange and respiratory mechanics, as assessed by an increase in PaO_2_/FiO_2_ of more than 20 mmHg or by a decrease in PaCO_2_ of more than 2 mm Hg, combined with an increase in dynamic lung compliance, for up to 24 h after intervention. Furthermore, we have aimed to determine the safety of bronchoscopy and predictors for clinical improvement.

## Material and methods

Design: A retrospective, single-center cohort study.

Setting: The ICU of an academic hospital in Amsterdam from January 2011 till July 2015.

Patients: Adult patients (>18 years old) who underwent a bronchoscopy because of atelectasis on CXR and in which the standard therapy failed. Standard care consisted of blind suctioning of the airways, airway nebulization (with bronchodilators and/or N-acetylcysteine), and chest physiotherapy. We excluded 28 patients whose exact time of bronchoscopy was lacking from the electronic medical record.

Procedure: Shortly before and during the bronchoscopy, the fractional inspired oxygen was raised to 100%. Mechanical ventilation was continued throughout the procedure in a volume controlled mode with an increased upper limit of maximum airway pressure. Sedation was provided as required and titrated by the intensive care physician. All bronchoscopies were performed by or under the direct supervision of a pulmonologist.

### Data and characteristics

Demographic information, patient characteristics, data on admission, and ventilation were collected for all patients from the electronic medical record system PDMS (Metavision®, IMD-soft, Tel-Aviv, Israel). The outcome variables were abstracted immediately before and 1, 12, and 24 h after the bronchoscopy.

Response to bronchoscopy was deemed as clinically relevant based on the improvement of oxygenation, as defined by an increase in the ratio of arterial oxygen partial pressure to fractional inspired oxygen (PaO_2_/FiO_2_ ratio) > 20 mmHg (12, 13), and an improvement of ventilation, defined as a decrease of > 2 mmHg in partial pressure of CO_2_ in arterial blood (PaCO_2_) (14). Pdriving was calculated as Peak pressure (Ppeak) − positive end expiratory pressure (PEEP), where Ppeak was used as a surrogate for plateau pressure, which was not available. Dynamic compliance was calculated as tidal volume divided by Ppeak. We defined pneumonia as the combination of positive microbiological culture, purulent sputum, and an infiltration on CXR.

The findings obtained during bronchoscopy were retrieved from bronchoscopy reports. Data on the location of atelectasis and pulmonary infiltrate were retrieved from CXR reports when available. The CXRs were evaluated by a staff radiologist. Radiographic improvement was defined as a resolution of atelectasis and/or increased aeration on CXR.

### Statistical analysis

At the start of the study protocol, we estimated that the study would be able to detect a clinically relevant improvement of oxygenation (PaO_2_/FiO_2_ ratio > 20 mmHg) after bronchoscopy (with α level of two-tailed test as 0.05) at a power of 80% if the total sample size was at least 98 cases.

Baseline patient characteristics were recorded and tabulated. Categorical variables were summarized using frequencies and percentages. Normally distributed continuous variables were summarized by mean ± standard deviation (± SD) and non-normally distributed variables by median and interquartile range (IQR).

To examine the longitudinal changes in continuous outcome variables after bronchoscopy, mixed linear models were used with time as the independent (categorical) variable. In case of a significant overall effect of time, *post-hoc* tests were used to compare means at each of the follow-up times separately with baseline measurement. Residuals were checked for normality. In case the normality assumption was violated, the nonparametric Friedman test followed by a *post-hoc* testing using Wilcoxon signed-rank testing was used.

Chi-square tests and univariate and multivariate logistic regression analyses were used to identify the predicting factors for clinical improvement after bronchoscopy, with additional ROC curve analysis to determine optimal cutoff points. Covariates included in the multivariable model were those associated with clinical improvement in univariate analyses at a significance level of *P* < 0.1, and a backward elimination method was used. *P* < 0.05 was considered as statistically significant. For *post-hoc* comparisons, a Bonferroni correction was used to account for the three separate comparisons of the follow-up measurements with the baseline. All *p*-values reported for the *post-hoc* tests have been corrected for multiple testing and should also be compared to the 0.05 significance level. All statistical analyses were performed using the IBM SPSS 20 statistical software package (SPSS Inc.®, Chicago USA).

## Results

### Patient characteristics

The characteristics of all patients are presented in Table 1. Between January 2011 and July 2015, 129 bronchoscopies were performed for the indication of atelectasis in 116 patients. Of these, 28 patients were excluded due to unsure and/or inadequate recording of bronchoscopy timing, making the total study population amount to 101 bronchoscopies in 88 patients. These 88 patients comprised of 7% of the total ICU population who required > 72 h of mechanical ventilation in the study period. At the time of bronchoscopy, most patients were mechanically ventilated (64%), 17% of the patients were ventilated noninvasively, and only 19% of the patients were without respiratory support.

**Table 1:** Baseline demographics and general outcome.

**Baseline demographics and general outcome**		**Data available on N (%)**
**INCLUDED PATIENTS** ***N*** = **88**		
APACHE2 score at admission^*^	23.2 (±10,7)	88 (100)
Age (years)^†^	63 [46–71]	88 (100)
Sex male/female^‡^	57/31 (65/35)	88 (100)
BMI (kg/m2)^*^	25.5 (±6,1)	88 (100)
Admission type/specialty	88 (100)	
Pulmonary Diseases^‡^	27 (30,7)	
Thoracic surgery^‡^	15 (17,0)	
General surgery^‡^	19 (21,5)	
Neurosurgery^‡^	13 (14,8)	
Miscellaneous^‡^	14 (15,9)	
Pulmonary history at time of admission^‡^	34 (38,6)	88 (100)
Duration of total ICU admission (days)^*^	20.6 (±17,4)	86 (98)
ICU mortality^‡^	20 (22,7)	88 (100)
Duration of mechanical ventilation (h)^†^	240 [80–514]	81 (92)
**INCLUDED BRONCHOSCOPIES** ***N*** = **101**		
Ventilation type^§^	101 (100)	
Without ventilator support^‡^	19 (18,8)	
Noninvasive ventilation^‡^	17 (16,8)	
Mechanical ventilation (intubated)^‡^	65 (64,4)	
Pressure control ventilation	41 (40,6)	
Pressure support ventilation	24 (23,7)	
Dynamic compliance (ml/cmH20)^†^^§^	29 [22–42]	70 (69)
Respiratory rate (respiration/minute)^*^^§^	22.0 (±9,5)	89 (88)
Heart rate (beats/minute)^*^^§^	95.1 (±18,3)	101 (100)
Inotropes ^‡^^§^	54 (53,5)	101 (100)
PaO_2_/FiO_2_ ratio (mmHg)^*^^§^	183.9 (±89,12)	82 (81)
PaCO_2_ (mmHg)^†^^§^	45 [40.5–53]	97 (96)

* Values are mean (±SD).

† Values are median [IQR].

‡ Values are N (%).

§ Baseline measurements were recorded 1 h before bronchoscopy.

*BMI, body mass index; APACHE 2, Acute Physiology and Chronic Health Evaluation II (15); PaO_2_/FiO_2_ ratio, the ratio of arterial oxygen partial pressure to fractional inspired oxygen; PaCO_2_, partial pressure of CO_2_ in arterial blood sample (mmHg)*.

### Bronchoscopy, radiology and side effects

The indication, main findings, and interventions during bronchoscopy are presented in Table 2. Atelectasis of one lobe (59%) was the main indication for bronchoscopy.

**Table 2:** Indication, main findings, interventions during bronchoscopy, and associated outcome and side effects.

**Findings during bronchoscopy and on chest X ray (CXR)**	**N (%)**	**Data available on N (%)**
Number of atelectatic lobes seen on CXR^*^		97 (96)
1	60 (59)	
2	31 (31)	
3	6 (6)	
>3	0 (0)	
Number of obstructed (secondary) bronchi found during bronchoscopy		101 (100)
0 (or not reported)	33 (33)	
1	30 (30)	
2	25 (25)	
3	10 (10)	
>3	3 (3)	
Bronchoscopic intervention		101 (100)
Intervention reported (airway suctioning of secretion)	90 (89)	
Reported obstruction of bronchi due to secretion	68 (67)	
Bronchoscopy not terminated early		101 (100)
Complications^§^	13 (13)	98 (97)
Desaturation between 80 and 90%	10 (10)	
Discomfort requiring additional sedation	1 (1)	
Hypotension due to sedation	1 (1)	
Arrhythmia	1 (1)	

Values are N (%) unless otherwise stated.

* As stated in CXR report. Four missing, atelectasis were seen on CT scan.

§ *Mild complications not requiring early termination of bronchoscopy, no severe complications found*.

In 89% of the cases, airway suctioning during bronchoscopy was reported. Obstruction due to this secretion was reported in 67% of the cases. Mostly, obstruction of one bronchus was seen (30%).

Subsequent CXR was performed in 45 subjects and improvement was recorded in 96% of the subjects.

Except for mild complications, occurring in 13% of the subjects, no adverse events were stated. Desaturation, with saturations between 80 and 90%, was encountered the most. None of the bronchoscopies were terminated early (Table 2).

### Gas exchange

Results of bronchoscopy on gas exchange are shown in Table 3. The PaO_2_/FiO_2_ ratio and measurements involving end tidal CO_2_ were calculated solely on invasively mechanical ventilated patients and respiratory mechanics solely on intubated and pressure control ventilated patients.

**Table 3:** Results of bronchoscopy on gas exchanges.

**Variable**	**Outcome**	**Difference to baseline**	***p-value***	**Subgroup analysis ± Outcome**	**Difference to baseline**	***p-value***
PaO_2_/FiO_2_ ratio overall			< 0.001			< 0.001
Baseline^*^	184 (±89)^†^			145 (±78.1)^†^		
1 h	214 (±100)^†^	30 (6.4 to 53.5)^‡^	0.007	182 (±92.5)^†^	37 (−0.2 to 74)^‡^	0.052
12 h	234 (±93)^†^	50 (25.7 to 73.4)^‡^	< 0.001	230 (±90.5)^†^	85 (48 to 123)^‡^	< 0.002
24 h	233 (±92)^†^	49 (24.0 to 73.1)^‡^	< 0.001	225 (±94.1)^†^	80 (42 to 118)^‡^	< 0.002
EtCO_2_			0.39			0.182
Baseline^*^	38.4 (±6.8)^†^			37.4 (±7.6)^†^		
1 h	37.4 (±7.6)^†^	−1 (−3.0 to 1.0)^‡^	0.669	35.3 (±7.4)^†^	−2.1 (−4.7 to 0.45)^‡^	0.143
12 h	38.7 (±8.3)^†^	0.3 (−1.6 to 2.3)^‡^	1.000	37.1 (±9.0)^†^	0 (−2.9 to 2.2)^‡^	1.000
24 h	38.4 (±7.7)^†^	0 (−2.0 to 2)^‡^	1.000	36.1 (±6.8)^†^	−1 (−4 to 1.4)^‡^	0.749
Art-etCO_2_ overall			< 0.001			0.001
Baseline^*^	11.0 (±9.5)^†^			12 (±10.5)^†^		
1 h	9.6 (±7.4)^†^	−1.4 (−3.4 to 0.6)^‡^	0.304	9.7 (±7.1)^†^	−2.6 (−5.4 to 0.3)^‡^	0.108
12 h	8.3 (±7.8)^†^	−2.7 (−4.8 to −0.6)^‡^	0.005	8.1 (±8.9)^†^	−4.1 (−7 to −1.2)^‡^	0.003
24 h	7.4 (±6.4)^†^	−3.6 (−5.7 to −1.4)^‡^	< 0.001	7.8 (±5.7)^†^	−4.5 (−7.4 to −1.4)^‡^	0.002
PaCO_2_ overall			0.057			0.006
Baseline^*^	46 [41–53]^§^			46 [41–53]^§^		
1 h	45 [41–53]^§^	1^#^	1.000	43 [40–50]^§^	3^#^	0.012
12 h	46 [41–52]^§^	0^#^	0.366	43 [38–47]^§^	3^#^	0.018
24 h	45 [40–50]^§^	1.5^#^	0.042	42 [37–47]^§^	4^#^	0.006

Bonferroni correction has already been applied to p-values for all post-hoc tests (follow-up vs. baseline).

PaO_2_/FiO_2_ ratio, measurements involving end tidal CO_2_ and respiratory mechanics were calculated solely on invasively mechanical ventilated patients.

± Subgroup analysis on intubated and pressure control ventilated patients (n = 41).

* Baseline measurements were recorded 1h before bronchoscopy. PaO_2_/FiO_2_ ratio, the ratio of arterial oxygen partial pressure to fractional inspired oxygen (mmHg); EtCO_2_, end tidal measurement of CO_2_ (mmHg); Art-etCO_2_, arterial pCO_2_ end tidal CO_2_ difference (mmHg), compliance (ml/cmH2O); PaCO_2_, partial pressure of CO_2_ in arterial blood sample (mmHg); PEEP, positive end expiratory pressure (cmH2O); Ppeak, peak pressure (cmH2O); Pdriving, Ppeak-PEEP (cmH2O).

† Mean (±SD).

‡ Mean difference compared to baseline (95% CI).

§ Median [IQR].

# *Median difference compared to baseline*.

Clinically relevant improvement of oxygenation was seen in 76% of the subjects and clinically relevant improvement of ventilation in 59% of the subjects. Both oxygenation and ventilation improved in 49% of the subjects.

A statistically significant increase in the PaO_2_/FiO_2_ ratio was seen 1, 12, and 24 h after bronchoscopy for all procedures. Arterial-end tidal CO_2_ difference decreased 12 and 24 h post bronchoscopy. No statistically significant difference was observed for end tidal measurement of CO_2_ (etCO_2_). When performing a subgroup analysis on pressure control ventilated patients, these observed differences persisted or became even larger. In case of PaCO_2_, a statistically significant decrease was found only in the subgroup analysis. No statistically significant changes in oxygenation, ventilation or respiratory mechanics were seen in the cases (representing 11% of the total) in which no intervention was performed during bronchoscopy.

### Respiratory mechanics

Respiratory mechanics were calculated solely on intubated and pressure control ventilated patients. Bronchoscopy improved compliance, Ppeak and Pdriving, while tidal volume remained the same, at 12 h post-bronchoscopy. These results persisted for at least 24 h after bronchoscopy. No statistically significant difference in PEEP was observed at any time after bronchoscopy, so respiratory mechanics improved without adjustment of PEEP (Table 4).

**Table 4:** Results of bronchoscopy on respiratory mechanics on subgroup intubated, pressure control ventilated patients (*n* = 41).

**Variable**	**Subgroup analysis ± Outcome**	**Difference to baseline**	***P*-value**
**Compliance (Dynamic) overall**			< 0.001
Baseline^*^	26 [20–30]^§^		
1 h	28 [22–34]^§^	2^#^	0.04
12 h	34 [27–47]^§^	8^#^	< 0.002
24 h	35 [27–99]^§^	9^#^	< 0.002
**Peep overall**			0.001
Baseline^*^	11 [8–15]^§^		
1 h	12 [8–16]^§^	1^#^	0.26
12 h	11 [8–14]^§^	0^#^	1.000
24 h	10 [8–14]^§^	−1^#^	0.138
**Ppeak overall**			< 0.001
Baseline^*^	29 [22–36]^§^		
1 h	28 [23–35]^§^	1^#^	1.200
12 h	26 [20–31]^§^	3^#^	0.003
24 h	26 [15–30]^§^	3^#^	< 0.002
**Pdriving overall (Ppeak-Peep)**			< 0.001
Baseline^*^	16 [14–21]^§^		
1 h	15 [13–20]^§^	1^#^	0.430
12 h	14 [10–16]^§^	2^#^	< 0.002
24 h	14 [6–16]^§^	2^#^	< 0.002

Bonferroni correction has already been applied to p-values for all post-hoc tests (follow-up vs. baseline).

^±^ Subgroup analysis on intubated and pressure control ventilated patients (n = 41).

* Baseline measurements were recorded 1 h before bronchoscopy. Compliance (ml/cmH2O), PEEP, positive end expiratory pressure (cmH2O); Ppeak, peak pressure (cmH2O); Pdriving, Ppeak-PEEP (cmH2O).

§ Median [IQR].

# *Median difference compared to baseline*.

**Figure 1:**
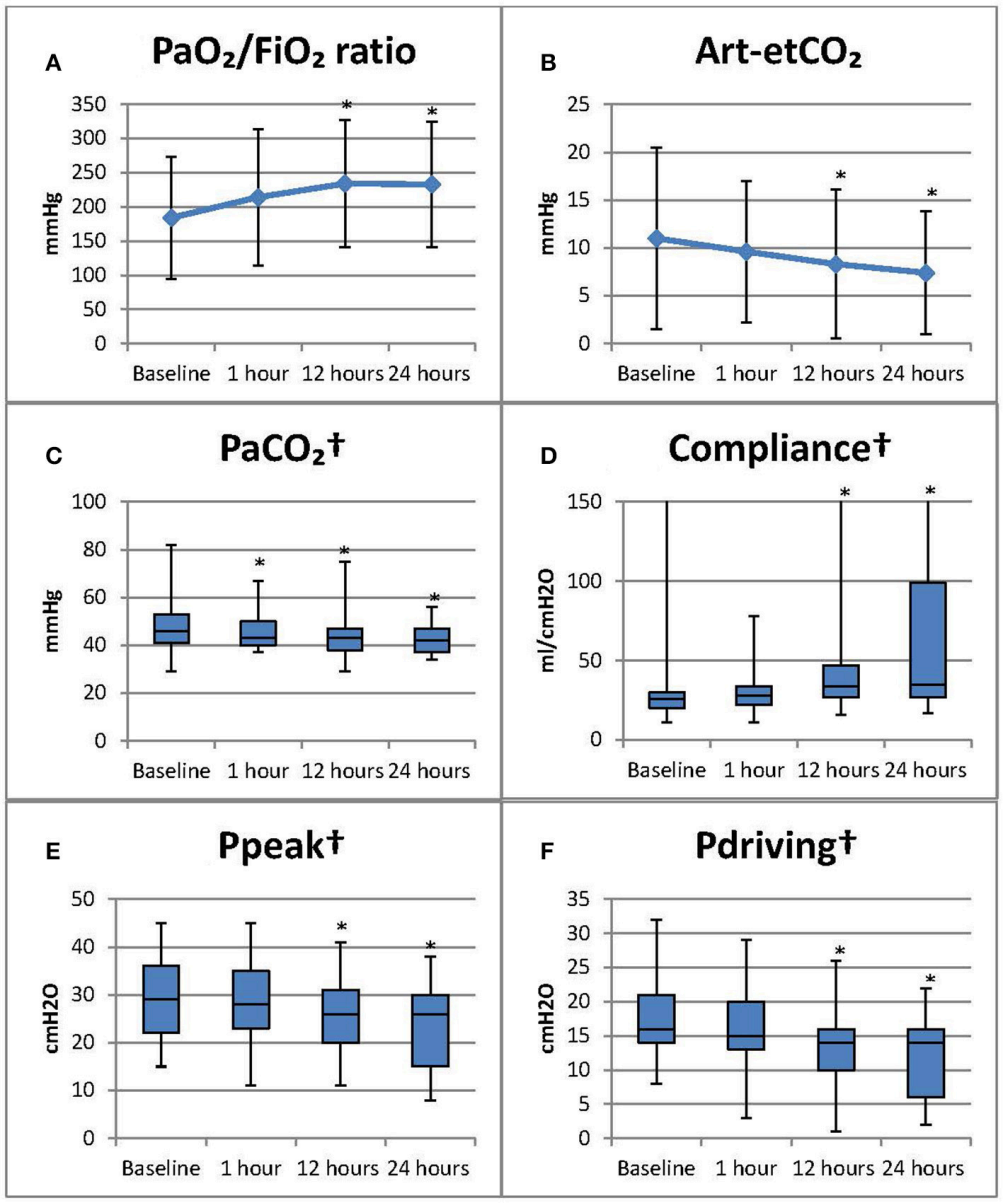
Results of bronchoscopy on gas exchange and respiratory mechanics *Statistically significant change compared to baseline (*p* < 0.05 Bonferroni correction has already been applied to *p*-values for all *post-hoc* tests, follow-up vs. baseline). Baseline measurements were recorded 1 h before bronchoscopy. Subsequent measurements, respectively at 1, 12, and 24 h post bronchoscopy. PaO_2_/FiO_2_ ratio, measurements involving end tidal CO_2_ were calculated solely on invasively mechanical ventilated patients. ^†^Results of subgroup analysis on intubated and pressure control ventilated patients. **(A)** PaO_2_/FiO_2_ ratio: the ratio of arterial oxygen partial pressure to fractional inspired oxygen (mmHg); **(B)** Art-etCO_2_: arterial pCO_2_ end tidal CO_2_ difference (mmHg); **(C)** PaCO_2_: partial pressure of CO_2_ in arterial blood sample (mmHg); **(D)** Compliance (ml/cmH2O); **(E)**. Ppeak: peak pressure (cmH2O); **(F)** Pdriving: Ppeak-PEEP (cmH2O), PEEP: positive end expiratory pressure (cmH2O). PaO_2_/FiO_2_ ratio and art-etCO_2_ presented as median [IQR]; all other parameters as mean (±SD).

All statistically significant changes in gas exchange and respiratory mechanics are shown in Figure 1.

### Predictors of successful bronchoscopy

To identify individual predictors for clinical improvement after bronchoscopy, univariate logistic regression analyses were performed on several baseline characteristics. Results are presented in Table 5.

**Table 5:** Univariable analysis of selected baseline characteristics on clinical improvement of oxygenation.

	**Clinical improvement of oxygenation OR (95% CI)**	***P*-value**	**Clinical improvement of ventilation OR (95% CI)**	***P*-value**	**Clinical improvement of oxygenation AND ventilation OR (95% CI)**	***P*-value**
**BASELINE CHARACTERISTICS**						
PaO_2_/FiO_2_ ratio	0.99 (0.98–0.99)	0.00	0.99 (0.99–1.00)	0.13	0.99 (0.99–1.00)	0.01
PaCO_2_	0.99 (0.95–1.04)	0.79	1.16 (1.08–1.25)	0.00	1.07 (1.02–1.13)	0.00
Compliance	0.99 (0.98–0.99)	0.04	0.99 (0.98–1.00)	0.08	0.99 (0.98–1.00)	0.08
Number of atelectic lobes^*^	1.57 (0.70–3.53)	0.27	1.04 (0.57–1.92)	0.89	1.27 (0.66–2.42)	0.48
Infiltration on CXR^*^	0.63 (0.21–1.90)	0.41	1.62 (0.60–4.37)	0.34	0.61 (0.22–1.68)	0.34
Purulent sputum	0.84 (0.29–2.47)	0.75	2.22 (0.82–5.99)	0.12	0.62 (0.25–1.56)	0.31
Bronchial toilet frequency^†^	0.98 (0.88–1.09)	0.72	1.02 (0.92–1.12)	0.74	1.02 (0.92–1.12)	0.73
Presence of pneumonia^‡^	1.35 (0.27–6.82)	0.72	1.82 (0.51–6.48)	0.36	0.83 (0.24–2.96)	0.78
Positive sputum culture	1.13 (0.41–3.10)	0.82	1.29 (0.57–2.96)	0.55	1.20 (0.52–2.77)	0.67

Clinical improvement on oxygenation is defined as PaO_2_/FiO_2_ ratio increase >20 mmHg (12, 13).

Clinical improvement on ventilation is defined as PaCO_2_ decrease > 2 mmHg (14).

PaO_2_/FiO_2_ ratio, the ratio of arterial oxygen partial pressure to fractional inspired oxygen (mmHg); PaCO_2_, partial pressure of CO_2_ in arterial blood sample (mmHg), dynamic compliance (ml/cmH2O); CXR, chest X ray.

* Number of atelectic lobes, infiltration as described in chest X-ray (CXR) reported before performance of bronchoscopy.

† Bronchial toilet frequency as performed in 12 h before bronchoscopy.

‡ *Presence of pneumonia was defined as the combination of purulent sputum, positive sputum culture, and an infiltrate seen on CXR*.

Of all the variables included, only baseline recordings of a lower PaO_2_/FiO_2_ ratio (OR 0.99 95% CI 0.99–1.00 *p* = 0.01) and higher PaCO_2_ (OR 1.07 95% CI 1.02,1.13 *p* < 0.01) were significantly associated with clinical improvement on both oxygenation and ventilation in the univariate analyses. In addition, low compliance (OR 0.99 95% CI 0.98–1.00 *p* = 0.08) was also included in a multivariate analysis. An association for clinical improvement on oxygenation was found for the baseline recordings of a low PaO_2_/FiO_2_ ratio (OR 0.99 95% CI 0.98–0.99 *p* < 0.01) and low compliance (OR 0.99 95% CI 0.98–1.00 *p* = 0.04). Additionally, high PaCO_2_ (OR 1.16 95% CI 1.08–1.25 *p* < 0.01) and low compliance (OR 0.99 95% CI 0.98–1.00 *p* = 0.08) were associated with clinical improvement on ventilation.

In the subsequent multivariable analyses, performed on a total of 70 cases due to missing data, only the baseline presence of high levels of PaCO_2_ remained as a predictive factor for clinical improvement of both oxygenation and ventilation (OR 1.14 95% CI 1.05–1.23 *p* = 0.001) and ventilation alone (OR 1.13 95% CI 1.05–1.23 *p* = 0.002). A baseline recording of low PaO_2_/FiO_2_ levels was the only remaining parameter that was significantly associated with clinical improvement on oxygenation (OR 0.991 95% CI 0.984–0.997 *p* = 0.006) (Table 6).

**Table 6:** Multivariable analysis of predictors for clinical improvement of oxygenation.

**Baseline characteristic**	**Clinical improvement on oxygenation OR (95% CI)**	***P*-value**	**Clinical improvement on ventilation OR (95% CI)**	***P*-value**	**Clinical improvement on oxygenation AND ventilation OR (95% CI)**	***P*-value**
PaO_2_/FiO_2_ ratio	0.991 (0.984–0.997)	0.006	1.00 (0.99–1.00)	0.198	1.00 (0.99–1.00)	0.135
PaCO_2_	1.006 (0.928–1.091)	0.885	1.13 (1.05–1.23)	0.002	1.14 (1.05–1.23)	0.001
Compliance	0.992 (0.982–1.002)	0.108	1.00 (0.98–1.00)	0.314	0.99 (0.98–1.01)	0.300

Clinical improvement on oxygenation is defined as PaO_2_/FiO_2_ ratio increase >20 mmHg (12, 13).

Clinical improvement on ventilation is defined as PaCO_2_ decrease > 2 mmHg (14).

*PaO_2_/FiO_2_ ratio, the ratio of arterial oxygen partial pressure to fractional inspired oxygen (mmHg); PaCO_2_, partial pressure of CO_2_ in arterial blood sample (mmHg), dynamic compliance (ml/cmH2O)*.

By analyzing PaO_2_/FiO_2_ ratio and PaCO_2_ in a receiver operating characteristic (ROC) curve optimal cutoff points were determined. For PaO_2_/FiO_2_ ratio, a baseline value of ≤ 210 mmHg is predictive of clinical improvement on oxygenation after bronchoscopy with a sensitivity of 70% and a specificity of 66%. PaCO_2_ as predictive of clinical improvement on ventilation alone or on oxygenation and ventilation, a cut-off value of ≥ 44,5 mmHg will render a sensitivity of 72% and specificity of 74% in our patient population.

## Discussion

The main results of this study are that bronchoscopy, when performed in the case of atelectasis in critically ill patients, was clinically beneficial in most patients by improving oxygenation (76%), ventilation (59%), or both (49%) for at least 24 h. In addition, in intubated and pressure control ventilated patients, a significant improvement was found in dynamic compliance, Ppeak, and Pdriving, with positive effects lasting for up to 24 h. Patients with a low baseline recording of PaO_2_/FiO_2_ and high baseline recording of PaCO_2_ seemed to benefit the most. Lastly, bronchoscopy was safe in these patients.

While previously published articles have been dominated by the effects of bronchoscopy on re-expansion of the collapsed pulmonary region on CXR (5–10), the clinical course will arguably be dictated more by respiratory mechanics. So, an improvement of long-term gas exchange and respiratory mechanics, as found in this study, will be of more clinical relevance than the aeration on CXR. When reviewing previously published studies that investigated the effects of bronchoscopy, it is noticed that most articles focus on short-term evaluation of gas exchange, finding no statistically significant results, and reported effects on respiratory mechanics are largely missing (4, 6, 16). Today, much emphasis is placed on lung-protective ventilation strategies to reduce ventilator-induced lung injury by maintaining alveolar aeration, preventing overexpansion of the lung, and limiting driving pressure (17). Our study is the first to assess respiratory mechanics after bronchoscopy in this lung protective ventilation perspective. Our results suggest that using bronchoscopy for atelectasis may be an addition to a lung protective strategy when considering the fact that bronchoscopy decreased Ppeak and Pdriving, as required for optimal gas exchange, with results lasting for at least 24 h. This finding merits further studies.

To date, there has been only one study investigating the effects of bronchoscopy on gas exchange and respiratory mechanics for up to 24 h. In Weinstein et al. (11) have shown a significant increase in the ratio of arterial to alveolar oxygen pressures (PaO_2_/PAO_2_) at 11 ± 1 h after lavage in 81% of the 43 lavages performed in only 6 patients; 63% of the 43 lavages were associated with a significant increase in effective static compliance at 8 ± 1 h after the lavage.

The long-term improvement of gas exchange might be offset by an initial deterioration after bronchoscopy, as stated by Jolliet and Chevrolet (16). This review of the literature concluded that gas exchange showed initial deterioration as measured by a PaCO_2_ increase of on average 8.25 mmHg and a PaO2 decrease of between 8.25 and 18.75 mmHg. This is probably being derived from smaller tidal volumes that are delivered while the bronchoscope is in place. When suctioning was applied, this effect became even larger; PaCO_2_ rose by 30%, while PaO2 decreased by about 40% due to reduced end-expiratory volume and PEEP. The authors stated that normalization takes 15 min in healthy lungs and up to hours in diseased lungs (16). This phenomenon, next to the time necessary for recovery of the ventilation-perfusion mismatch by mitigating hypoxic pulmonary vasoconstriction, can explain that we found no improved gas exchange 1 h after the procedure.

Although the risk of losing lung volume during or after bronchoscopy exists, by either derecruitment, denitrogenation, or negative pressure into the respiratory system, less atelectasis was seen on subsequent CXR (18).

Bronchoscopy can be considered safe in ICU patients, considering the low complication rate found in our study, which is comparable to several previous publications (10, 19, 20). The most recent and largest publication on safety of bronchoscopy in mechanically ventilated ICU patients with sepsis, septic shock, and/or acute lung injury/acute respiratory distress syndrome prospectively collected data on 100 mechanically ventilated patients undergoing bronchoscopy with bronchial lavage. They found an overall complication rate of 10%, with hypoxemia during or immediately after the procedure being the most frequently encountered complication (9%) and with only one bronchoscopy being terminated early (20).

The strengths of our study are in the use of the largest cohort in literature for examining the effects of bronchoscopy for the sole indication of atelectasis and on clinical relevant outcomes, as well as the long term follow-up, for up to 24 h post intervention. Furthermore, this is the first study to investigate predictors for the clinical improvement of gas exchange.

There are a number of limitations to this study, the most important being its observational retrospective design and inherent bias, resulting in an inability to control for confounders. No data is available on pulmonary recruitment before or after the procedure. This decision was left to the clinical judgment of the attending intensive care physician. Also, the true incidence of adverse events may be underestimated in a retrospective study. Furthermore, a suboptimal calculation was used to calculate Pdriving. Missing data at one point of time after intervention is also a limitation in our study. By dismissing the incomplete case series, the inclusion of cases for nonparametric testing ranged between 57 and 85%.

Current evidence on this topic has been dominated by observational studies that investigate the short-term effects of bronchoscopy. Although our results suggest clinical improvement of gas exchange and pulmonary mechanics, future research should confirm our results in a prospective manner, preferably by a randomized controlled study. Subsequently research should focus on patient outcomes such as length of stay and ventilator-free days.

## Conclusion

In conclusion, bronchoscopy for atelectasis is beneficial in most ICU patients by improving gas exchange and mechanical properties of the respiratory system, with positive effects lasting for up to 24 h. In addition, bronchoscopy is safe in these patients.

## Availability of research data

Permission to publish the datasets supporting the conclusions of this article was not granted by our institution. Requests to access the datasets should be directed to the corresponding author (k.smeijsters@vumc.nl).

## Ethics approval and consent to participate

The research project was reviewed and approved by the medical ethics board at VUmc (reference 2017.084); informed consent was waived and de-identified patient data was used.

## Author contributions

KS had full access to all the data in the study and takes responsibility for the integrity of the data and the accuracy of the data analysis, including and especially any adverse effects, and was also responsible for manuscript preparation and revision. RB was involved in study design and data collection and revised the manuscript for important intellectual content. JD, AG, and LH were involved in study design and revised the manuscript for important intellectual content. PvdV was responsible for statistical analysis and manuscript revision. JS and PT supervised the project and were involved in all aspects of the study. All authors read and approved the final version of the manuscript.

### Conflict of interest statement

The authors declare that the research was conducted in the absence of any commercial or financial relationships that could be construed as a potential conflict of interest.

## Abbreviations

ICU: Intensive care Unit
CXR: Chest X- ray
Art-etCO_2_: arterial pCO_2_ end tidal CO_2_ difference
EtCO_2_: end tidal measurement of CO_2_
PaCO_2_: partial pressure of CO_2_ in arterial blood
PaO_2_/FiO_2_ ratio: the ratio of arterial oxygen partial pressure to fractional inspired oxygen
PEEP: positive end expiratory pressure
Pdriving: driving pressure (Ppeak-PEEP)
Ppeak: peak pressure.
